# Direct tracheostomy in Guillain-Barré syndrome: indication and timing both matter. Authors’ reply

**DOI:** 10.1016/j.aicoj.2026.100177

**Published:** 2026-10-01

**Authors:** Arnaud W. Thille, Louis Chamblet, Sylvain Le Pape, Rémi Coudroy

**Affiliations:** a CHU de Poitiers, Service de Médecine Intensive Réanimation, Poitiers, France; b University of Poitiers, INSERM CIC 14-02, IS-ALIVE Research Group, France

Dear Editor,

We are honored to answer questions about our recently published study in *Annals of Intensive Care* journal on ventilator weaning strategies in 494 patients intubated for Guillain–Barré syndrome [1]. In this post-hoc analysis of a large observational study [2], patients having undergone direct tracheostomy without any prior weaning attempt were less likely to be liberated from the ventilator at day 28 than those having undergone a standard weaning process involving spontaneous-breathing trials, a finding suggesting that direct tracheostomy may significantly delay the ventilator weaning process. According to Lin et al. [3], tracheostomy is typically performed in the event of persistent muscle weakness requiring prolonged mechanical ventilation or severe bulbar weakness requiring prolonged airway protection. However, the issue is not whether a tracheostomy is beneficial or harmful, but rather whether an early direct tracheostomy strategy is more harmful than a standard weaning process involving weaning attempts, with tracheostomy performed only later, if necessary. Although patients selected for direct tracheostomy may have more severe disease, it is difficult to predict which ones will experience major weaning difficulties and how quickly they will recover [4]. Therefore, a ventilator weaning strategy involving routine early direct tracheostomy could lead to unnecessary tracheostomy and extend the weaning process. We agree with the methodological concerns raised by C. Jiang [5], especially insofar as the time-zero point differed between the two groups, as direct tracheostomy occurred earlier than the initial weaning attempt. In other words, the decision to attempt an initial spontaneous-breathing trial reflects a certain degree of clinical recovery. Moreover, residual confounding factors may remain, even after adjustment using G-computation, particularly regarding variation in tracheostomy practices across the 47 participating centers. Indeed, the caterpillar plot (supplemental data) shows high heterogeneity in the weaning process across the different centers; while standard weaning was more commonly used in some centers, direct tracheostomy predominated in others. This suggests that the decision to perform a direct tracheostomy was not necessarily based on patient severity alone. Furthermore, we observed that direct tracheostomy was not associated at any center with better outcomes, suggesting that standard weaning may be preferable. As direct tracheostomy was performed earlier than standard weaning, this inherently introduces an immortal time bias (patients who undergo tracheostomy cannot subsequently undergo standard weaning). However, the proportion of patients liberated from the ventilator 28 days after intubation was also lower with direct tracheostomy than with standard weaning (Fig. 1). Immortal time bias also arises when comparing direct tracheostomy with tracheostomy after weaning failure, since weaning failure occurs much later. Nevertheless, patients having undergone tracheostomy after weaning failure are, by definition, the most difficult to wean, and even when compared with this group, direct tracheostomy was less effective in terms of ventilator liberation. Despite the retrospective nature of the study introducing numerous biases, we believe there is no signal supporting early direct tracheostomy without any attempt at weaning. Tracheostomy should only be considered after sufficient time has elapsed to confirm the need for prolonged mechanical ventilation, persistent bulbar dysfunction, and after weaning attempts.

**Fig. 1:**
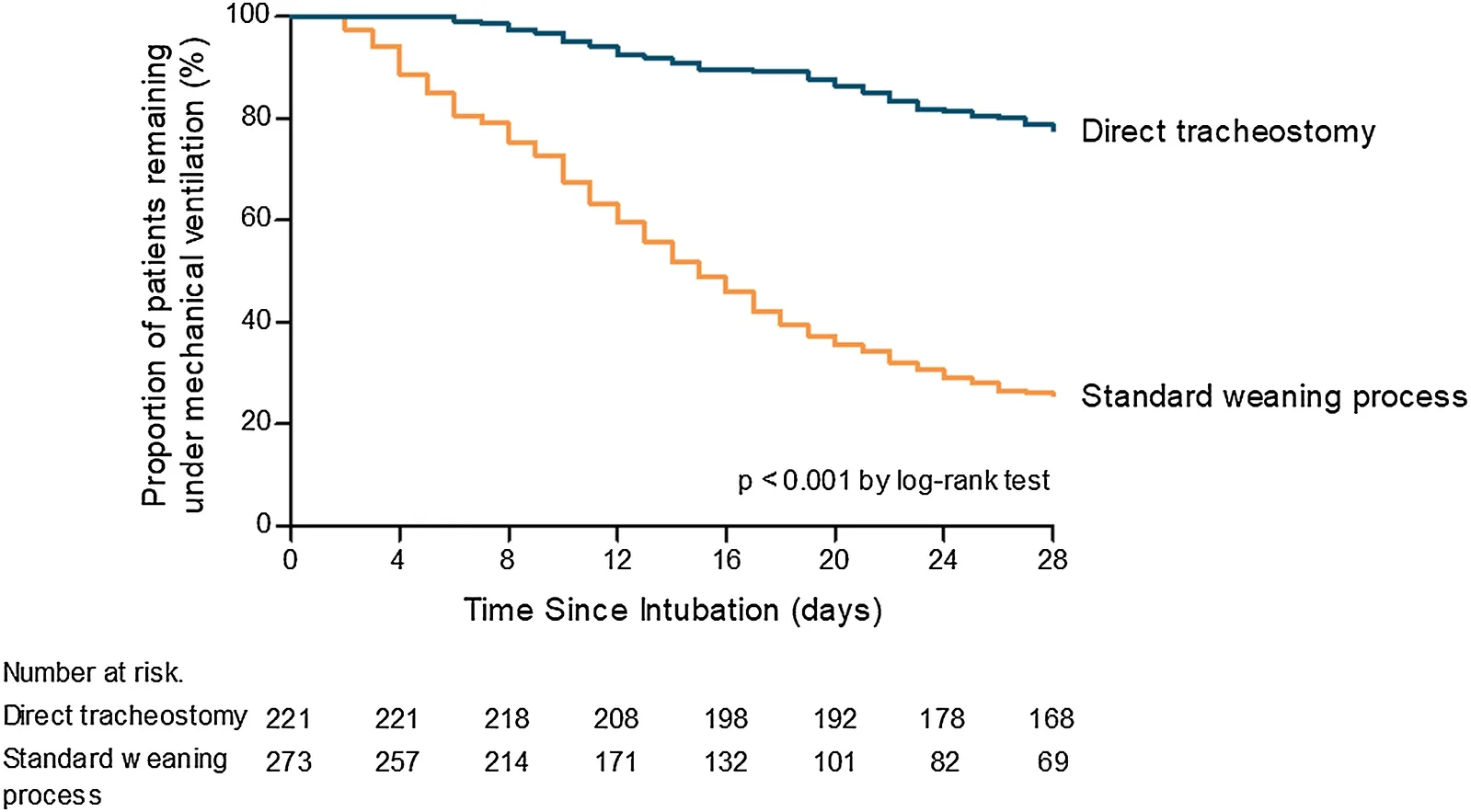
Kaplan–Meier curves showing the probability of remaining under mechanical ventilation within the 28 days following intubation. The proportion of patients alive and free from mechanical ventilation within the 28 days following intubation was significantly higher with standard weaning (orange curve) than with direct tracheostomy (blue curve) (74% vs. 22%, p < 0.001 using log-rank test).

## Funding source

None.
